# Kir4.2 Potassium Channels in Retinal Pigment Epithelial Cells In Vitro: Contribution to Cell Viability and Proliferation, and Down-Regulation by Vascular Endothelial Growth Factor

**DOI:** 10.3390/biom12060848

**Published:** 2022-06-18

**Authors:** Marie-Christin Beer, Heidrun Kuhrt, Leon Kohen, Peter Wiedemann, Andreas Bringmann, Margrit Hollborn

**Affiliations:** 1 Department of Ophthalmology and Eye Hospital, University of Leipzig, 04103 Leipzig, Germany; marie.ch.beer@gmail.com (M.-C.B.); leon.kohen@helios-gesundheit.de (L.K.); peter.wiedemann@medizin.uni-leipzig.de (P.W.); bria@medizin.uni-leipzig.de (A.B.); 2 Institute of Anatomy, University of Leipzig, 04103 Leipzig, Germany; heidrun.kuhrt@medizin.uni-leipzig.de; 3 Department of Ophthalmology, Helios Klinikum Aue, 08280 Aue, Germany

**Keywords:** retinal pigment epithelium, Kir4.2, hypoxia, hyperosmolarity, cell proliferation, cell viability, VEGF

## Abstract

Dedifferentiation and proliferation of retinal pigment epithelial (RPE) cells are characteristics of retinal diseases. Dedifferentiation is likely associated with changes of inwardly rectifying potassium (Kir) channels. The roles of Kir4.2 channels in viability, and proliferation of cultured RPE cells were investigated. Gene expression levels were determined using qRT-PCR. RPE cells expressed *Kir2.1*, *2.2*, *2.4*, *3.2*, *4.1*, *4.2*, *6.1*, and *7.1* mRNA. Kir4.2 protein was verified by immunocytochemistry and Western blotting. *Kir4.2* mRNA in cultured cells was upregulated by hypoxia (hypoxia mimetic CoCl_2_ or 0.2% O_2_) and extracellular hyperosmolarity (addition of high NaCl or sucrose). *Kir4.2* mRNA was suppressed by vascular endothelial growth factor (VEGF), blood serum, and thrombin whereas platelet-derived growth factor (PDGF), basic fibroblast growth factor (bFGF), and transforming growth factor-β1 (TGF-β1) increased it. Hyperosmotic *Kir4.2* gene expression was mediated by TGF-β1 receptor signaling while hypoxic gene transcription was dependent on PDGF receptor signaling. VEGF receptor-2 blockade increased *Kir4.2* mRNA level under control, hyperosmotic, and hypoxic conditions. SiRNA-mediated knockdown of Kir4.2 decreased the cell viability and proliferation under control and hyperosmotic conditions. Kir4.2 channels play functional roles in maintaining the viability and proliferation of RPE cells. Downregulation of Kir4.2 by VEGF, via activation of VEGF receptor-2 and induction of blood-retinal barrier breakdown, may contribute to decreased viability of RPE cells under pathological conditions.

## 1. Introduction

The metabolic, structural, and functional support of photoreceptors by the retinal pigment epithelium (RPE) includes the homeostasis of the subretinal fluid [1]. Maintenance of the subretinal space volume and ion and osmohomeostasis of the subretinal fluid are pivotal for regular photoreceptor activity. There is also a fluid transport from the subretinal space to the choroidea that is a water flux across the RPE which is osmotically coupled to ion fluxes through transporter and channel molecules [2,3]. At the apical membranes of RPE cells, a large potassium conductance is the major pathway for potassium in- and efflux which mediates the spatial buffering of the subretinal potassium concentration, supports the activities of the Na^+^–K^+^ pump and Na^+^–K^+^–2Cl^−^ cotransporter, and governs the resting membrane potential [4]. RPE cells express a diversity of inwardly rectifying potassium (Kir) channel subtypes [5] which mediate larger inward than outward potassium currents [6]. Diverse Kir channel subtypes are expressed in distinct membrane domains. The apical processes which surround the photoreceptor segments contain Kir4.1 channels that likely mediate potassium buffering of the subretinal fluid whereas the roots of the apical processes express Kir7.1 channels; the potassium efflux through Kir7.1 supports the activity of the Na^+^-K^+^ pump [7]. 

The physiological properties of RPE cells change in eyes with diabetic retinopathy, age-related macular degeneration (AMD), and proliferative vitreoretinopathy. RPE cells become activated and less differentiated (epithelial-mesenchymal transition), lose the monolayer arrangement, proliferate, and migrate through the neuroretina and choroid [8,9,10,11]. Hyperreflective foci in various retinal and choroidal layers on spectral-domain optical coherence tomography images of patients with diverse retinal diseases were suggested to partly represent migrating RPE cells [11,12,13,14,15,16]. The frequency of hyperreflective foci correlates with disease progression (e.g., with the development of geographic atrophy in AMD eyes that is associated with a degeneration of the RPE and photoreceptors) [11,13,17,18]. Transdifferentiated RPE cells are a main source of vascular endothelial growth factor (VEGF) [8,19] which is the principal hypoxia-induced angiogenic factor implicated in the development of diabetic retinopathy and neovascular AMD, for example [20]. Suppressing the dedifferentiation process was shown to greatly reduce the severity of proliferative vitreoretinopathy [21]. 

Activation and dedifferentiation of RPE cells are associated with alterations in cell physiology; a more detailed understanding of the alterations is pivotal for the finding of new therapeutic targets [11]. The alterations of the physiological properties of RPE cells under pathological conditions are likely associated with changes of membrane channel expression (e.g., of Kir channels). It was shown that the *Kir4.1* gene expression in RPE cells of the rat is downregulated in experimental retinal vein occlusion [22] and that the Kir4.1 immunoreactivity disappears after RPE cell isolation [7]. It is likely that Kir channels serve different functional roles in differentiated and activated RPE cells. In a first attempt to investigate this assumption, the expression of diverse Kir channel genes in human RPE cells were compared between acutely isolated (differentiated) and cultured cells. Subconfluently cultured cells can be considered as in-vitro model of dedifferentiated RPE cells with physiological properties similar to activated RPE cells in situ [11,23]. 

We found in the Kir channel gene screening that acutely isolated and cultured cells express, among other Kir channel transcripts, *Kir4.1* (*KCNJ10*) and *Kir4.2* (*KCNJ15*) gene transcripts. In a further step, we investigated the regulation of the expression of *Kir4.1* and *Kir4.2* genes in cultured cells by pathogenic conditions and diverse growth and blood factors. We found that the expression of both genes is upregulated in hypoxia and that the *Kir4.2* gene expression is also upregulated in response to alterations of the extracellular osmolarity. We examined the effect of extracellular hyperosmolarity induced by high NaCl since hypertension is a risk factor of diabetic retinopathy and AMD [24,25,26,27]; the main cause of acute increases of the blood pressure is hypernatremia following intake of dietary salt (NaCl) [28,29]. Hypernatremia induces systemic hyperosmolarity [30,31] that is associated with blood volume expansion and hypertension [29,32]. In an attempt to investigate the functional roles of Kir4.2 channels in activated RPE cells, we determined the effects of a knockdown of Kir4.2 with small interfering RNA (siRNA) on the gene expression and secretion of VEGF, and the viability and proliferation of cultured RPE cells.

## 2. Materials and Methods

### 2.1. Materials

Cell culture components and solutions were purchased from Gibco BRL (Paisley, UK). Human recombinant vascular endothelial growth factor (VEGF-A_165_), pigment epithelium-derived factor (PEDF), transforming growth factor-β1 (TGF-β1), platelet-derived growth factor (PDGF)-BB, insulin-like growth factor (IGF), basic fibroblast growth factor (bFGF), hepatocyte growth factor (HGF), heparin-binding epidermal growth factor-like growth factor (HB-EGF), and epidermal growth factor (EGF) were obtained from R&D Systems (Abingdon, UK). Human recombinant placental growth factor-2 (PlGF-2) was purchased from Reliatech (Braunschweig, Germany). Human recombinant activated blood coagulation factor X (FXa), was obtained from Calbiochem (Bad Soden, Germany). The inhibitor of hypoxia-inducible transcription factor (HIF)-1, caffeic acid phenethyl ester (CAPE), 666-15, SU1498, and SR11302 were provided by Tocris (Ellisville, MO, USA). Stattic and AG1478 were obtained from Enzo Life Science (Lausen, Switzerland). PD173074 was provided by Pfizer (Karlsruhe, Germany). Human-specific siRNA against nuclear factor of activated T cell 5 (NFAT5; sc-43968) and Kir4.2 (sc-91419), respectively, as well as nontargeted scrambled siRNA (sc-37007) were purchased from Santa Cruz Biotechnology (Heidelberg, Germany). Human recombinant α-thrombin, 1,10-phenanthroline, actinomycin D, tetracaine, AG1296, SB431542, and all other agents used were purchased from Sigma-Aldrich (Taufkirchen, Germany), unless stated otherwise. 

### 2.2. Cell Culture

Eyes of human cornea donors without reported eye disease (12 females, mean ± S.D. age, 60.1 ± 21.5 years; 15 males, mean age, 57.7 ± 19.2 years) were received within 48 h of death with the written informed consent from the relatives for the use of retinal tissue in basic science. There were no significant (*p* > 0.05) differences between data obtained with cells from younger and aged donor eyes, and with cells from both sexes (not shown). RPE cells were prepared and cultured as described [33]. Cultured cells of passages 3–5 were used. Cells which achieved a confluence of approximately 90% were cultured 16 h in serum-free medium. During this period, 100% confluence was achieved. Thereafter, test substances were applied to the serum-free medium. Hyperosmotic media were prepared by administration of NaCl or sucrose. Extracellular hypoosmolarity was established by addition of distilled water. Hypoxia was established by cell culture in a 0.2% O_2_-atmosphere; chemical hypoxia was induced by adding 150 µM of the hypoxia mimetic CoCl_2_ which stabilizes HIF-1α and -2α in normoxia [34,35]. Inhibitory agents were applied 30 min before the begin of the tests. 

### 2.3. RNA Extraction and cDNA Synthesis

Total RNA was extracted using the InviTrap Spin Universal RNA Mini Kit (Stratec Molecular, Berlin, Germany). The A_260_/A_280_ ratio of the optical density of the RNA samples was determined using NanoDrop1000 (peQLab, Erlangen, Germany), and was between 1.95 and 2.05 which indicates adequate RNA quality. Following the use of DNase I (Roche, Mannheim, Germany), cDNA was synthesized from 0.25 µg RNA using a reverse transcription kit (Thermo Fisher Scientific, Waltham, MA, USA). 

### 2.4. RT-PCR Analysis

RT-PCR was performed using the Taq PCR Master Mix kit (Qiagen, Hilden, Germany); primer pairs are given in Table 1. The transcripts were amplified for 40 cycles in a volume of 20 µL, containing 1 µL of the first-strand mixture and 0.25 µM of primer pairs, using the PTC-200 Thermal Cycler (MJ Research, Watertown, MA, USA); the amplification conditions were 30 s at 94 °C, 60 s at 58 °C or 60 °C (*Kir4.2*), and 1 min at 72 °C. 

### 2.5. Real-Time RT-PCR Analysis (qRT-PCR)

Real-time RT-PCR was carried out using the CFX Connect Real-Time PCR System (BioRad, Munich, Germany) with primer pairs described in Table 1. The amplification mixture (10 μL) contained 5 μL of 2× iQ SYBR Green Supermix (BioRad), specific primer set (0.2 µM each), and 3 µL (75 ng; *Kir4.2*) or 1 μL (25 ng; *ACTB*, *β2M*, and all other genes investigated) of cDNA. The amplification conditions were: denaturation at 95 °C for 3 min (one cycle), 45 cycles denaturation (95 °C, 30 s), annealing (58 °C or 60 °C for *Kir4.2*; 20 s), extension (72 °C; 45 s), and melting curve (increasing temperature from 55 °C to 95 °C in 0.5 °C steps). The correct length of PCR products was proved using agarose gel electrophoresis. The mRNA levels were normalized to the level of *ACTB* mRNA or β2-microglobulin (*B2M*) mRNA (in the Kir4.2 siRNA experiments) [36]. Relative mRNA expression levels were estimated according to the 2^−^^ΔΔCT^ method [37]. The PCR efficiencies for *ACTB* (104%), *B2M* (102%), *Kir4.2* (105%), *NFAT5* (103%), and *VEGFA* (101%) were similar in the examined range between 0.73 and 220 ng of cDNA.

### 2.6. siRNA Transfection

Transfection of NFAT5 siRNA (5 nM), Kir4.2 siRNA (10 nM), or nontargeted scrambled siRNA, was performed using Lipofectamine RNAiMAX (Invitrogen, Paisley, UK) in F-10 medium with 10% fetal bovine serum (FBS; Invitrogen). The medium was changed after 48 h. Fresh medium without FBS or containing 0.5% FBS (proliferation and viability assays) with NaCl (+100 mM) or CoCl_2_ (150 µM) was added for further 24 h. Total RNA was extracted, and the *Kir4.2*, *NFAT5*, and *VEGFA* mRNA levels or the proliferation rate and viability of the cells was determined. 

### 2.7. Immunocytochemistry

Cultures were fixed 15 min on ice with 4% paraformaldehyde. After washing with prechilled phosphate-buffered saline (PBS; pH 7.4; Invitrogen), PBS containing 0.3% Triton X-100 was applied 15 min at room temperature (RT). Nonspecific antibody binding was blocked for 2 h at RT by PBS which contained 10% normal goat serum and 0.3% Triton X-100. The primary antibody diluted in blocking solution was incubated at 4 °C overnight. After washing with PBS which contained 0.3% Triton X-100, the secondary antibody was incubated 1 h at RT. After further washing steps, 4′,6-diamidin-2-phenylindol (DAPI; 1:10,000.; Invitrogen) was given for 15 min at RT. The coverslips were mounted using Fluorescence Mounting Medium (DakoCytomation, Glostrup, Denmark). The images were recorded using Olympus BX40 (Olympus, Essex, UK) with a CCD camera (Olympus XM10) and the OLYMPUS cellSens Dimension 1.16 software (Olympus, Essex, UK). Antibodies used are described in Table 2. 

### 2.8. Western Blot Analysis

Protein extracts from acutely isolated neuroretinas of two donors were prepared using the Mammalian Cell Lysis kit (MCL-1; Sigma-Aldrich). In order to prepare cytosolic (soluble proteins) and membrane protein extracts of cultured cells, cells were cultured in 75-cm^2^ culture flasks in F-10 medium containing 10% FBS until confluence was reached. After removal of the medium, the cells were washed with prechilled PBS and detached from the culture flasks with a cell scraper. After centrifugation, the cells were lysed in buffer A (0.5 mL) which contained 50 mM Tris-HCl (pH 7.4), 150 mM NaCl, 1 mM EDTA, 1% protease inhibitor cocktail (Sigma-Aldrich), 1% phosphatase inhibitor cocktail (Sigma-Aldrich), and 0.5% phenylmethylsulfonyl fluoride. The lysates were centrifuged at 14,000 rpm (15 min; 4 °C), and the supernatants (containing soluble cytosolic proteins) were removed and used for Western blotting; the remaining pellets (containing the membrane fraction of the cells) were solubilized in 400 µL of buffer B that contained 50 mM Tris-HCl (pH 7.4), 150 mM NaCl, 1 mM EDTA, 1% Triton X-100, 0.1% SDS, 1% protease inhibitor cocktail, and 1% phosphatase inhibitor cocktail. 

In order to prepare whole cell protein extracts, cells were cultured in 6-well plates. After removal of the medium, the cells were washed with prechilled PBS and scraped into lysis buffer (180 µL) that contained 50 mM Tris-HCl (pH 8.0), 5 mM EDTA, 150 mM NaCl, 0.5% phenylmethylsulfonyl fluoride, 0.5% NP-40 detergent solution (Thermo Fisher Scientific), 1% protease inhibitor cocktail, and 1% phosphatase inhibitor cocktail. The lysates were centrifuged at 14,000 rpm (10 min; 4 °C). Equal amounts of protein (30 µg) were separated by 12.5% SDS-polyacrylamide gel electrophoresis. Primary and secondary antibodies (Table 2) were applied, and immunoreactive bands were visualized with 5-bromo-4-chloro-3-indolyl phosphate/nitro blue tetrazolium. 

### 2.9. ELISA, Cell Proliferation and Viability Assays

siRNA-transfected and nontransfected cells were maintained 24 h in medium without or with 0.5% FBS (cell proliferation and viability assays) in the presence or absence of high (+100 mM) NaCl or 150 µM CoCl_2_. The level of VEGF-A_165_ in the culture supernatants (200 µL) was determined with ELISA (R&D Systems). Incorporation of bromodeoxyuridine (BrdU) was measured using the Cell Proliferation ELISA BrdU Kit (Roche, Mannheim, Germany). BrdU (10 μM) was applied 5 h before culture fixation. Cell viability was evaluated using a 3-(4,5-dimethylthiazol-2-yl)-2,5-diphenyltetrazolium bromide (MTT) assay (Serva, Heidelberg, Germany). MTT solution (10 µL; 5 mg/mL) was given to each well 4 h before cessation of cell culture. Culture supernatants were removed and dimethylsulfoxide (100 µL) was added. The absorbance at 570 nm was measured using Spectra Max 50 (Molecular Devices, Sunnyvale, CA, USA). 

### 2.10. Statistical Analysis

For each test, at least three independent experiments using cells of different donors were carried out. Data are presented as means ± S.E.M. Statistical analysis was performed using the Prism 6 Version 6.07 program (Graphpad Software, San Diego, CA, USA). Statistical significance was evaluated using one-way ANOVA followed by Bonferroni’s multiple comparison test as well as Mann-Whitney *U* test, and was accepted at *p* < 0.05.

## 3. Results

### 3.1. Kir Channel Gene Expression in Human RPE Cells 

**Figure 1:**
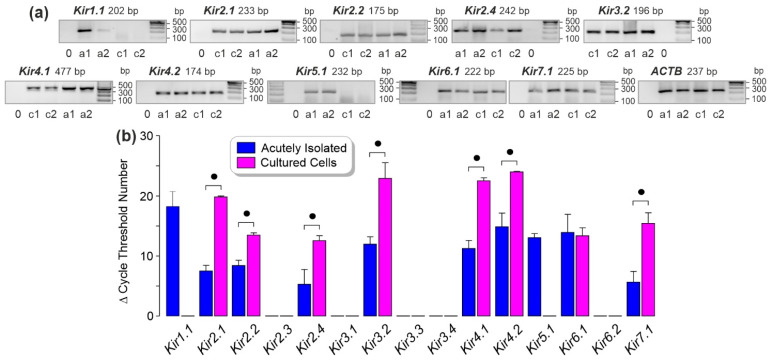
Kir channel gene expression in human RPE cells. (**a**) Agarose gel electrophoresis performed with products of acutely isolated (a) and cultured cells (c) from different post-mortem donors. 0, negative control carried out by the addition of double-distilled water instead of cDNA as a template. The level of β-actin (*ACTB*) mRNA was used to normalize the Kir channel mRNA levels in real-time RT-PCR analysis. (**b**) Comparison of the expression levels of Kir channel genes in acutely isolated and cultured RPE cells. The data were evaluated by real-time RT-PCR. The bars show the cycle numbers at the threshold of transcript detection. Cells derived from 4 (cultured cells) to 6 eyes (acutely isolated cells) of different donors were used. Significant difference between acutely isolated and cultured cells: ● *p* < 0.05.

RT-PCR analysis was carried out to compare the expression of diverse Kir channel genes in RPE cells which were acutely isolated from eyes of post-mortem human donors (differentiated cells) and cultured RPE cells (dedifferentiated cells). Both acutely isolated and cultured cells contained transcripts of the following Kir channel genes: *Kir2.1*, *Kir2.2*, *Kir2.4*, *Kir3.2*, *Kir4.1*, *Kir4.2*, *Kir6.1*, and *Kir7.1* (Figure 1a). Transcripts of *Kir1.1* and *Kir5.1* genes were detected in acutely isolated, but not in cultured cells (Figure 1a). The levels of the following Kir channel transcripts were below the detection threshold in both acutely isolated and cultured cells: *Kir2.3*, *Kir3.1*, *Kir3.3*, *Kir3.4*, and *Kir6.2* (not shown). The levels of *Kir2.1*, *Kir2.2*, *Kir2.4*, *Kir3.2*, *Kir4.1*, *Kir4.2*, and *Kir7.1* transcripts were smaller in cultured than acutely isolated cells, as shown by the significantly (*p* < 0.05) higher cycle numbers required to detect the transcripts in cultured cells (Figure 1b). The cycle numbers necessary to detect *Kir6.1* transcripts were not significantly (*p* > 0.05) different between acutely isolated and cultured cells (Figure 1b). The mRNA expression levels are shown in the Supplemental Figure S1. 

### 3.2. Kir4.2 Protein in Cultured RPE Cells

**Figure 2:**
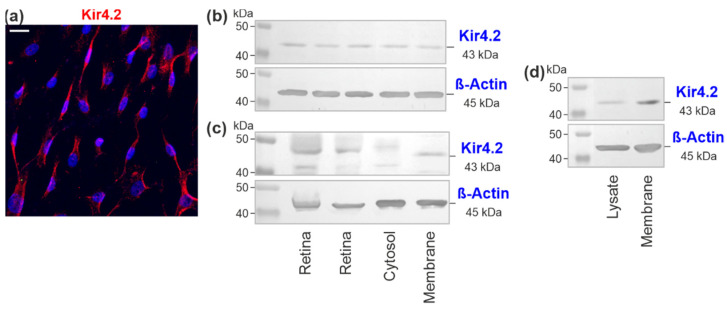
Kir4.2 protein in cultured human RPE cells. (**a**) Immunolabeling of a cell culture with an anti-Kir4.2 antibody (*red*). Cell nuclei were stained with DAPI (*blue*). Bar, 20 µm. (**b**) Western blot analysis of Kir4.2 protein performed with lysates of cells from five donors. (**c**) Western blots of Kir4.2 protein performed with lysates of acutely isolated neuroretinas from two post-mortem donors (*left side*) and cytosolic and membrane protein extracts of cells from one donor (*right side*). (**d**) Western blot of Kir4.2 protein performed with lysate and membrane protein extract of cells from one donor. (**a**,**c**,**d**) The images show one example of three experiments carried out using cells of different donors. The following antibodies were used for western blotting: rabbit anti-human Kir4.2 (**c**) and mouse anti-human Kir4.2 (**b**,**d**).

Cultured RPE cells showed Kir4.2 immunoreactivity with a punctate pattern predominantly along cell processes (Figure 2a). Western blots of cell lysates displayed a Kir4.2-immunoreactive band around 43 kDa (Figure 2b) which was not present in control blots (data not shown). The presence of Kir4.2 protein in lysates of acutely isolated neuroretinas of two donors was determined as positive control (Figure 2c). When cytosolic (soluble proteins) and membrane proteins were extracted from the cells, Western blot analysis revealed the presence of an immunoreactive band of Kir4.2 protein in the membrane protein, but not cytosolic extract (Figure 2c). Accordingly, western blots of whole cell lysates and membrane protein extracts showed a more intensely stained immunoreactive band in the blot of the membrane proteins compared to the blot of the cell lysate (Figure 2d). The data indicate that Kir4.2 protein is predominantly localized to membranes of RPE cells. 

### 3.3. Regulation of Kir4.1 and Kir4.2 Gene Expression by Pathogenic Conditions

**Figure 3:**
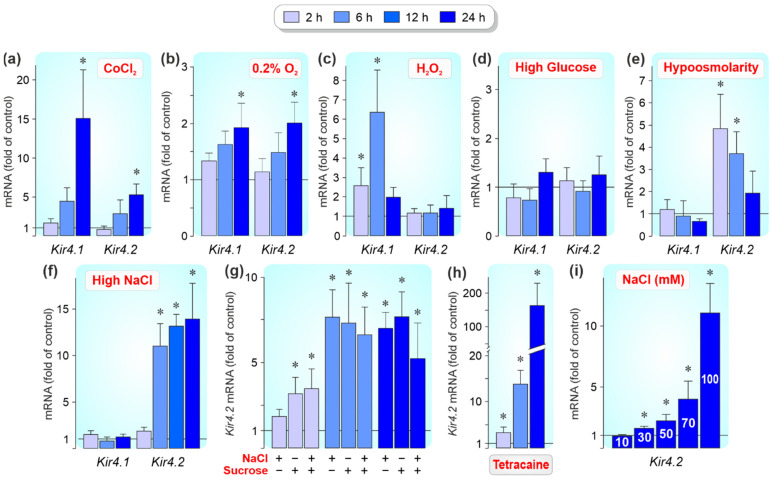
Regulation of *Kir4.1* and *Kir4.2* gene expression in cultured RPE cells by pathogenic conditions. Real-time RT-PCR was carried out with RNA extracted from cells which were stimulated 2, 6, and 24 h. In (**f**), a 12-h stimulation was also examined on *Kir4.2* gene expression. Relative mRNA levels are shown as folds of unstimulated control (1). (**a**) Effects of chemical hypoxia, induced by adding 150 µM CoCl_2_, on the Kir channel gene expression. (**b**) Effect of cell culture in a 0.2% O_2_-atmosphere. (**c**–**f**) Effects of oxidative stress (20 µM H_2_O_2_; (**c**)), hyperglycemia (25 mM glucose; (**d**)), extracellular hypoosmolarity (60% osmolarity; (**e**)), and hyperosmolarity (+100 mM NaCl; (**f**)). (**g**) Effects of hyperosmotic media prepared by adding 100 mM NaCl or 200 mM sucrose, or by coaddition of NaCl and sucrose, on the *Kir4.2* gene expression. (**h**) Effect of tetracaine (1 mM) on the *Kir4.2* mRNA level. (**i**) Dose-dependent effect of high NaCl on the *Kir4.2* mRNA level. Different concentrations (10–100 mM) of NaCl were added to the culture media. Each bar shows data derived from 3–9 independent experiments with cells of different donors. Significant difference vs. unstimulated control: * *p* < 0.05.

We tested different agents to screen which pathogenic conditions alter the expression levels of *Kir4.1* and *Kir4.2* genes in cultured RPE cells. The hypoxia mimetic CoCl_2_ [34,35] induced significant (*p* < 0.05) increases of the cellular levels of *Kir4.1* and *Kir4.2* transcripts after 24 h of stimulation (Figure 3a). The stimulatory effect of hypoxia on the *Kir4.1* and *Kir4.2* gene expression was confirmed in cells cultured in a 0.2% O_2_-atmosphere which also induced significant (*p* < 0.05) increases of the gene expression after 24 h of stimulation (Figure 3b). Oxidative stress produced by adding H_2_O_2_ caused an increase of the *Kir4.1* gene expression after 2 and 6 h of stimulation, and did not alter the *Kir4.2* mRNA level (Figure 3c). High (25 mM) glucose did not alter the *Kir4.1* and *Kir4.2* gene expression within 24 h of stimulation (Figure 3d). Extracellular hypoosmolarity induced an increased expression of the *Kir4.2* gene after 2 and 6 h of stimulation while extracellular hyperosmolarity (produced by adding 100 mM NaCl) induced an increase of the *Kir4.2* gene expression after 6, 12, and 24 h of stimulation; both treatments had no effect on the expression of the *Kir4.1* gene (Figure 3e,f). 200 mM sucrose, which caused an equal increase in osmolarity as 100 mM NaCl, induced a similar increase of the *Kir4.2* mRNA level as addition of 100 mM NaCl after 6 and 24 h of stimulation (Figure 3g). Coaddition of sucrose and NaCl did not result in a higher *Kir4.2* mRNA level after 6 and 24 h of stimulation (Figure 3g), suggesting that high NaCl induced *Kir4.2* gene expression by the increase of the extracellular osmolarity and not by the change of the NaCl gradient across the plasma membrane. Sucrose, but not high NaCl, induced a significant (*p* < 0.05) increase of the *Kir4.2* gene expression after 2 h of stimulation (Figure 3g). The reason for this difference is unclear; it could be that the cells are capable to partly compensate the altered NaCl gradient across the plasma membrane after 2 h, but not after 6 and 24 h of stimulation. It cannot be ruled out that Kir4.2 channels are involved in the regulation of the cellular sodium homeostasis since the sodium channel blocker tetracaine [38] caused a high increase in the cellular *Kir4.2* mRNA level which was time-dependent and highest after 24 h of stimulation (Figure 3h). High NaCl induced a dose-dependent increase in the level of *Kir4.2* transcripts; after 24 h of stimulation, significant (*p* < 0.05) increases were found after the addition of more than 10 mM NaCl (Figure 3i). 

**Figure 4:**
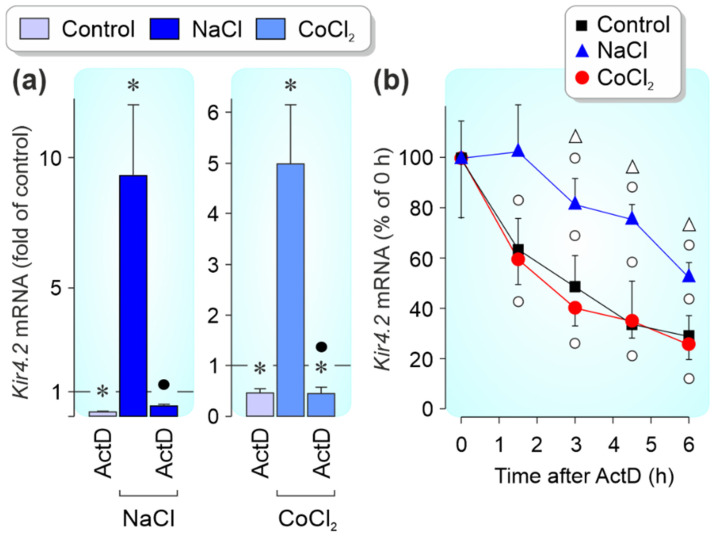
*Kir4.2* gene transcription and mRNA stability. The hyperosmotic and CoCl_2_-induced expression of the *Kir4.2* gene in cultured RPE cells is mediated by stimulation of gene transcription; extracellular hyperosmolarity also induces an increase of *Kir4.2* mRNA stability. The relative mRNA level is shown as fold of unstimulated control (1; (**a**)) and percent of the 0-h control (**b**). (**a**) The NaCl- and CoCl_2_-induced *Kir4.2* gene expression was prevented by actinomycin D (ActD; 5 µg/mL) which blocks RNA polymerase II. The cells were cultured 12 h in the absence and presence of high (+100 mM) NaCl and 24 h in the absence and presence of CoCl_2_ (150 µM), respectively. (**b**) The stability of the *Kir4.2* mRNA differed significantly (*p* < 0.05; ∆) between cells cultured in the presence of high NaCl compared to cells cultured under control conditions and stimulated by CoCl_2_, respectively. The cells were first stimulated for 12 h with high (+100 mM) NaCl, and 24 h with CoCl_2_ (150 µM), respectively. Thereafter, actinomycin D (5 µg/mL) was added, and total RNA was isolated at different times. Each bar represents data obtained in 3–7 independent experiments using cells of different donors. Significant difference vs. unstimulated control: * *p* < 0.05. Significant difference vs. NaCl and CoCl_2_ control, respectively: ● *p* < 0.05. Significant difference vs. 0-h control: ○ *p* < 0.05.

**Figure 5:**
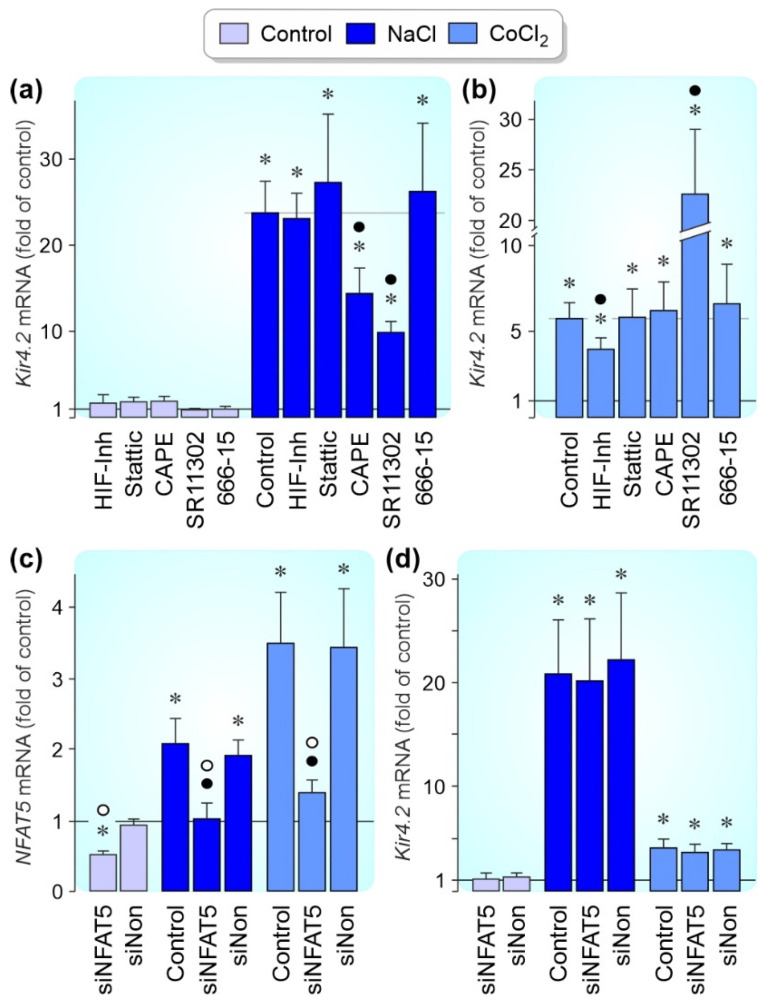
Activities of transcription factors which mediate the hyperosmotic and CoCl_2_-induced expression of the *Kir4.2* gene in cultured RPE cells. mRNA levels were examined with real-time RT-PCR analysis. Relative mRNA levels are shown as fold of unstimulated control (1). (**a**,**b**) The cells were maintained 24 h in the absence (control) and presence of high (+100 mM) NaCl (**a**) and CoCl_2_ (150 µM; (**b**)), respectively. The effects of a HIF-1 inhibitor (HIF Inh; 5 µM), the STAT3 inhibitor Stattic (1 µM), the NF-κB inhibitor CAPE (5 µM), the AP-1 inhibitor SR11302 (5 µM), and the CREB inhibitor 666-15 (250 nM) were tested. (**c**) Transfection of the cells with NFAT5 siRNA (siNFAT5; 5 nM) resulted in a reduction of the *NFAT5* mRNA level in RPE cells cultured 24 h in the absence (control) and presence of high (+100 mM) NaCl and CoCl_2_ (150 µM), respectively. Nontargeted scrambled siRNA (siNon; 5 nM) had no effects. (**d**) Knockdown of *NFAT5* gene expression with siRNA (siNFAT5; 5 nM) did not alter the *Kir4.2* mRNA level in cells cultured under the three conditions tested. Each bar represents data obtained in 3–7 independent experiments using cells of different donors. Significant difference vs. unstimulated control: * *p* < 0.05. Significant difference vs. NaCl and CoCl_2_ control, respectively: ● *p* < 0.05. Significant difference between siNFAT5 and siNon: ○ *p* < 0.05.

The NaCl-induced *Kir4.2* gene expression was mediated by induction of gene transcription (Figure 4a) and an increase of mRNA stability (Figure 4b). The CoCl_2_-induced increase of the *Kir4.2* mRNA level was mediated by induction of gene transcription (Figure 4a) while the mRNA stability was not different to cells cultured under control conditions (Figure 4b). The hyperosmotic *Kir4.2* gene expression (measured after 24 h of stimulation) was significantly (*p* < 0.05) suppressed by inhibitors of nuclear factor (NF)-κB (CAPE) [39] and activator protein (AP)-1 (SR11302) and not altered by inhibitors of HIF-1 [40], signal transducer and activator of transcription 3 (STAT3; Stattic) [41], and the cAMP response element-binding protein (CREB; 666-15) (Figure 5a). The CoCl_2_-induced *Kir4.2* gene expression (measured after 24 h of stimulation) was significantly (*p* < 0.05) suppressed by the HIF-1 inhibitor and elevated in the presence of the AP-1 inhibitor SR11302 (Figure 5b). 

NFAT5 is a transcription factor that is required for the survival of different cell systems in hyperosmotic conditions [42]; NFAT5 expression is increased in RPE cells in response to high extracellular NaCl but not to hypoxic conditions [43]. RPE cells which were transfected with NFAT5 siRNA showed a reduction of the NFAT5 mRNA level by approximately 50% compared to nontransfected cells and cells transfected with nontargeted siRNA 48 h after transfection (*p* < 0.05; Figure 5c); however, transfection of NFAT5 siRNA had no effect on the *Kir4.2* mRNA level under all conditions tested (Figure 5d). 

### 3.4. Regulation of Kir4.2 Gene Expression by Growth Factors

**Figure 6:**
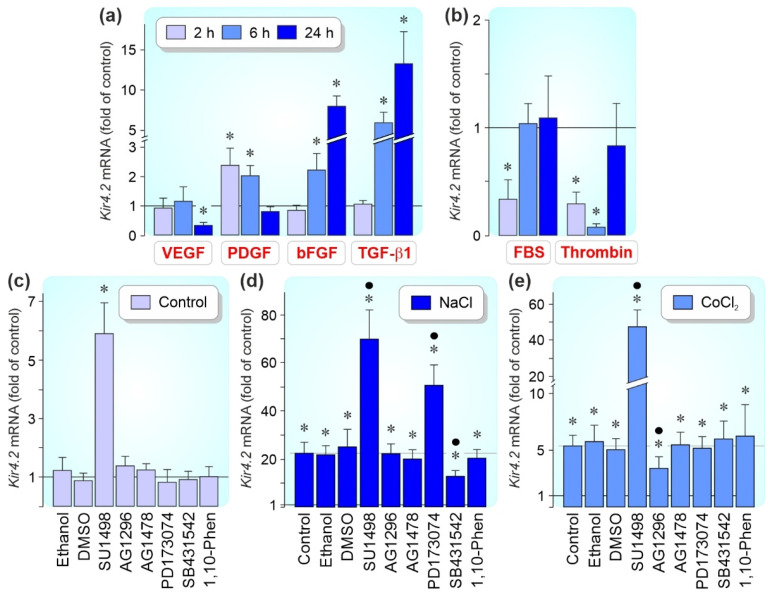
Effects of growth and blood factors on the *Kir4.2* gene expression in cultured RPE cells. (**a**,**b**). Real-time RT-PCR was performed with total RNA extracted from cells which were stimulated 2, 6, and 24 h. The relative mRNA level is shown as fold of unstimulated control (1). (**a**) Effects of exogenous VEGF, PDGF, bFGF, and TGF-β1 (each at 10 ng/mL) on the *Kir4.2* transcript level in cells cultured under control conditions. (**b**) Effects of fetal bovine serum (FBS; 10%) and thrombin (10 U/mL) under control conditions. (**c**–**e**) Effects of receptor blockers on the *Kir4.2* gene expression in cells cultured 24 h in the absence (**c**) and presence of high (+100 mM) NaCl (**d**) and CoCl_2_ (150 µM; (**e**)), respectively. The following compounds were applied: the inhibitor of the VEGF receptor-2, SU1498 (10 µM), the blocker of the PDGF receptor tyrosine kinase, AG1296 (10 µM), the EGF receptor tyrosine kinase inhibitor AG1478 (600 nM), the inhibitor of the FGF receptor kinase, PD173074 (500 nM), the blocker of TGF-β1 superfamily activin receptor-like kinase receptors, SB431542 (10 µM), and the broad-spectrum metalloproteinase inhibitor 1,10-phenanthroline (1,10-Phen; 10 µM). The effects of ethanol (1:100) and dimethylsulfoxide (DMSO; 1:500) are shown as vehicle controls. Each bar represents data obtained in 3–8 independent experiments using cells of different donors. Significant difference vs. unstimulated control: * *p* < 0.05. Significant difference vs. NaCl and CoCl_2_ control, respectively: ● *p* < 0.05.

The effect of different cytokines was investigated to reveal whether the expression of the *Kir4.2* gene in cultured RPE cells is modulated by growth factors. As shown in Figure 6a, exogenous VEGF caused a time-dependent decrease of the *Kir4.2* gene expression which was apparent after 24 h of stimulation whereas PDGF, bFGF, and TGF-β1 induced increases of the *Kir4.2* transcript level at different time periods (PDGF: at 2 and 6 h; bFGF and TGF-β1: at 6 and 24 h of stimulation). FBS (10%) caused a transient downregulation at 2 h of stimulation, and thrombin caused a downregulation at 2 and 6 h of stimulation (Figure 6b). The following factors induced no or slight changes of the *Kir4.2* mRNA level within 24 h after administration (not shown): EGF, HB-EGF, HGF, IGF, PlGF, PEDF (each at 10 ng/mL), and FXa (1 U/mL). 

It was shown that hyperosmotic conditions stimulate the secretion of growth factors including bFGF, VEGF, and TGF-β1 from cultured RPE cells [43,44,45]. By using selective receptor blockers, we investigated whether autocrine/paracrine activation of growth factor receptors contributes to the hyperosmotic and CoCl_2_-induced *Kir4.2* gene expression in RPE cells. The hyperosmotic *Kir4.2* gene expression (measured after 24 h of stimulation) was significantly (*p* < 0.05) reduced by inhibition of TGF-β1 superfamily activin receptor-like kinase receptors with SB431542 (Figure 6d). Blockers of the PDGF receptor tyrosine kinase (AG1296) and the EGF receptor tyrosine kinase (AG1478) were without effects (Figure 6d). In addition, the blocker of metalloproteinase activation, 1,10-phenanthroline, had no effect (Figure 6d), suggesting that a metalloproteinase-mediated release of growth factors from the extracellular matrix is not involved in mediating the hyperosmotic expression of the *Kir4.2* gene. 

The inhibitors of VEGF receptors-2, SU1498, and FGF receptor kinases, PD173074, increased significantly (*p* < 0.05) the *Kir4.2* mRNA level under hyperosmotic conditions; SU1498 also increased the *Kir4.2* expression level under control conditions (Figure 6c,d). The CoCl_2_-induced expression of the *Kir4.2* gene (measured after 6 h of stimulation) was suppressed by the blocker of the PDGF receptor tyrosine kinase, AG1296, and strongly increased by the blocker of the VEGF receptor-2, SU1498 (Figure 6e). The data indicate that autocrine/paracrine activation of VEGF receptors causes a downregulation of the *Kir4.2* gene expression under control, hyperosmotic, and chemical hypoxia conditions while the hyperosmotic expression of the *Kir4.2* gene is also suppressed by autocrine/paracrine activation of FGF receptors. 

### 3.5. Effects of Kir4.2 Knockdown on Cell Proliferation and Survival

**Figure 7:**
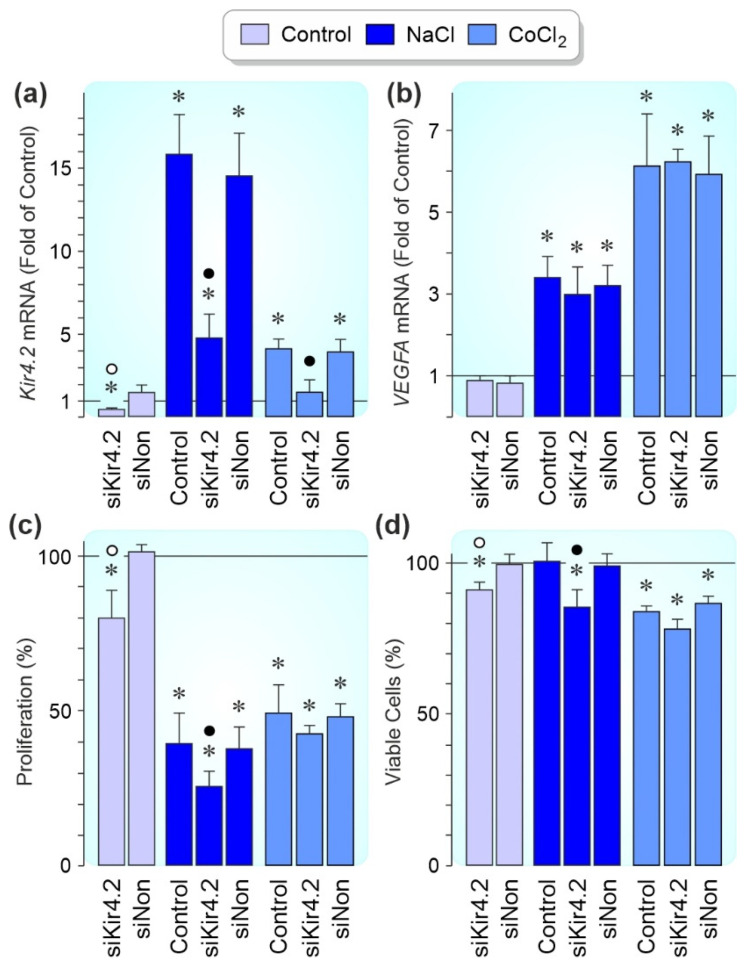
Kir4.2 knockdown affects the proliferation and viability of cultured RPE cells. The expression levels of the *Kir4.2* gene (**a**) and the *VEGFA* gene (**b**), the proliferation rate (**c**), and the viability (**d**) were investigated in cells transfected with Kir4.2 siRNA (siKir4.2) and nontargeted siRNA (siNon). The cells were cultured 24 h in the absence and presence of high (+100 mM) NaCl and CoCl_2_ (150 µM), respectively, as indicated by the panels of the bars. The data are shown as fold (1; (**a**,**b**)) and percent of unstimulated, nontransfected control cells (100%; (**c**,**d**)), respectively. Each bar represents data obtained in 4–10 independent experiments using cells of different donors. Significant difference vs. unstimulated, nontransfected control cells: * *p* < 0.05. Significant difference vs. NaCl and CoCl_2_ control, respectively, and transfection with siNon: ● *p* < 0.05. Significant difference between siKir4.2 and siNon: ○ *p* < 0.05.

In order to test whether Kir4.2 channels modulate the proliferation and viability of RPE cells, Kir4.2 was knocked down by siRNA. Transfection of Kir4.2 siRNA reduced significantly (*p* < 0.05) the level of *Kir4.2* mRNA by about 70% in cells cultured under control, hyperosmotic, and chemical hypoxia conditions (measured after 24 h of stimulation with high NaCl and CoCl_2_, respectively); transfection of nontargeted scrambled siRNA had no effects (Figure 7a). We found no effects of Kir4.2 knockdown on the expression of the *VEGFA* gene (Figure 7b) and the VEGF-A protein content of the media (not shown) of cells cultured 24 h in the absence and presence of high (+100 mM) NaCl and CoCl_2_ (150 µM), respectively. 

In different cell systems (e.g., RPE cells), extracellular hyperosmolarity was described to induce cell cycle arrest [46,47]. As previously shown [48], RPE cells cultured in the presence of high NaCl or CoCl_2_ displayed a decreased proliferation rate compared to cells cultured under control conditions (measured after 24 h of stimulation; Figure 7c). Under control and hyperosmotic conditions, the proliferation rate of Kir4.2 siRNA-transfected cells was significantly (*p* < 0.05) stronger decreased compared to cells transfected with scrambled siRNA and nontransfected cells (Figure 7c). This suggests that Kir4.2-mediated potassium currents are required to maintain cell proliferation under these conditions. The transfection of Kir4.2 siRNA also induced a slight, but not significant decrease of the cell proliferation rate under conditions of CoCl_2_-induced chemical hypoxia (Figure 7c). 

As previously described [45], the hypoxia mimetic CoCl_2_ decreased the RPE cell viability (measured after 24 h of stimulation; Figure 7d). Under control and hyperosmotic conditions, the viability of cells transfected with Kir4.2 siRNA was significantly (*p* < 0.05) lower compared to cells transfected with nontargeted scrambled siRNA and nontransfected cells (Figure 7d). Transfection of Kir4.2 siRNA was also associated with a slight, but nonsignificant suppression of the cell viability under conditions of CoCl_2_-induced chemical hypoxia (Figure 7d). The data suggest that Kir4.2 channels are necessary for the full viability of RPE cells. 

## 4. Discussion

We found in accordance with a previous study [5] that acutely isolated (differentiated) RPE cells express a diversity of Kir channel genes (Figure 1a). The expression levels of 9 genes were reduced in cultured cells compared to acutely isolated cells (Figure 1b). The cause for the downregulation in cultured cells is not clear. Since hypoxia induced *Kir4.1* and *Kir4.2* gene expression (Figure 3a), it cannot be ruled out that acutely isolated cells suffered from post-mortem hypoxia. A similar phenomenon was found in rat RPE cells which decrease their Kir4.1 immunoreactivity after isolation [7]. It may be possible that factors of the cellular environment in situ (e.g., the interphotoreceptor matrix and the activity-related alterations of the potassium concentration in the subretinal fluid) promote the channel expression in RPE cells. The differences in the expression levels of certain Kir channel genes may indicate that these channels play distinct roles in differentiated and activated (dedifferentiated) RPE cells. 

We observed that the expression of *Kir4.1* and *Kir4.2* genes in cultured RPE cells was increased in hypoxia (Figure 3a) and differentially regulated under other pathogenic conditions. Oxidative stress caused an upregulation of *Kir4.1*, but not *Kir4.2* gene expression (Figure 3c) while alterations of the extracellular osmolarity induced *Kir4.2*, but not *Kir4.1* gene expression (Figure 3e,f). The different regulation of *Kir4.1* and *Kir4.2* gene expression under various conditions may suggest that both channel subtypes play diverse functional roles in activated RPE cells. Kir4.2-mediated potassium currents are likely implicated in mediating the osmotic balance across the plasma membrane. The different kinetics of *Kir4.2* gene expression induced by extracellular hypo- and hyperpermeability (Figure 3e, f) may be caused by diverse adaptive mechanisms which allow the cells to survive under the different osmotic conditions. 

We found that receptor-mediated signalling regulates the expression of the *Kir4.2* gene under various conditions. Although exogenous bFGF stimulated the *Kir4.2* gene expression under control conditions (Figure 6a), blockade of the FGF receptor kinase by PD173074 increased the *Kir4.2* mRNA level in extracellular hyperosmolarity (Figure 6d); this suggests that endogenous bFGF has an inhibitory effect on the expression of the *Kir4.2* gene in extracellular hyperosmolarity. TGF-β1 stimulated the *Kir4.2* gene expression under control (Figure 6a) and hyperosmotic conditions (Figure 6d), and was without effect under CoCl_2_-induced hypoxic conditions (Figure 6e). Exogenous PDGF increased the *Kir4.2* gene expression under control conditions (Figure 6a) and the action of endogenous PDGF contributed to the hypoxic, but not hyperosmotic, expression of the *Kir4.2* gene (Figure 6d,e). 

RPE cells are a source of VEGF [8,19]. Both hypoxia and extracellular hyperosmolarity are known to stimulate the expression and secretion of VEGF in RPE cells [43]. We observed that exogenous VEGF caused a decrease of the *Kir4.2* gene expression in cultured RPE cells (Figure 6a) and that a selective blocker of VEGF receptor-2 (SU1498) increased the *Kir4.2* mRNA level under control, hyperosmotic, and chemical hypoxia conditions (Figure 6c–e). The latter suggests that autocrine/paracrine VEGF signaling suppresses the *Kir4.2* gene expression under these conditions. We also observed that blood factors (i.e., FBS and thrombin) decreased the *Kir4.2* gene expression (Figure 6b). Since VEGF is a factor which triggers a breakdown of the blood-retinal barrier (e.g., in neovascular AMD and diabetic retinopathy) [20], the data may suggest that VEGF induces a downregulation of Kir4.2 in RPE cells via two mechanisms: by a direct VEGF receptor-mediated effect and by an indirect effect through the breakdown of the blood-retinal barrier which permits a flux of blood constituents into the retina. Downregulation of Kir4.2 by VEGF may contribute to decreased viability of RPE cells under pathological conditions (e.g., to the pathogenesis of geographic atrophy in AMD eyes). 

Activated RPE cells in retinal diseases such as AMD, diabetic retinopathy, and proliferative vitreoretinopathy are characterized by altered physiological properties which include decreased viability, cell scattering, proliferation, and migration through the retinal and choroidal tissues [8,9,10,11,12,13,14,15,16]. We found that knockdown of Kir4.2 decreased the proliferation rate and viability of cultured RPE cells under control and hyperosmotic conditions (Figure 7c,d). Kir4.2 knockdown also decreased slightly both parameters under CoCl_2_-induced hypoxic conditions (Figure 7c,d). The data suggest that Kir4.2 channels play a role in the maintenance of the viability and proliferation of RPE cells. RPE cells express a variety of Kir channel genes (Figure 1a,b); therefore, it cannot be ruled out that the effects of Kir4.2 knockdown are partly compensated by the activities of other Kir channel types. In many cell systems, potassium channels play crucial roles in the regulation of cell proliferation and viability [49,50]. Potassium channels are implicated in the signal transduction evoked by cell-cycle checkpoints [49]. By keeping the resting membrane potential at hyperpolarized values [4], Kir channels provide the precondition for the regular activity of voltage-dependent sodium and calcium channels and the driving force for calcium influx; the latter is important for the calcium-dependent control of the cell cycle [51]. The strong increase of the cellular level of *Kir4.2* mRNA induced by the sodium channel blocker tetracaine (Figure 3h) might reflect a functional relationship between Kir4.2 channels and voltage-gated sodium channels. Further research is needed to identify the mechanisms how Kir4.2 channels contribute to the maintenance of RPE cell proliferation and viability. 

## 5. Conclusions

Human RPE cells express a diversity of Kir channel genes; the expression levels of seven genes are decreased in cultured (dedifferentiated) cells compared to acutely isolated (differentiated) cells. The expression of *Kir4.1* and *Kir4.2* genes in cultured cells is upregulated in hypoxia; *Kir4.2* gene expression is also stimulated by alterations of the extracellular osmolarity. The expression of the *Kir4.2* gene is regulated by growth and blood factors; VEGF, FBS, and thrombin decrease the *Kir4.2* gene expression while PDGF, bFGF, and TGF-β1 increase the expression. Autocrine/suppparacrine VEGF receptor-2 signaling also inhibits the expression of the *Kir4.2* gene under hyperosmotic and CoCl_2_-induced hypoxic conditions. siRNA-mediated knockdown of Kir4.2 causes reduction of the viability and proliferation of cultured RPE cells, suggesting a role of the channels in the maintenance of these physiological parameters. It is suggested that downregulation of Kir4.2 by VEGF may contribute to decreased viability of RPE cells under pathological conditions. 

## Figures and Tables

**Table 1:** Primer pairs used in PCR experiments. ^a^, primers used in RT-PCR analysis. ^b^, primers used in real-time RT-PCR analysis. s, sense. as, anti-sense.

Gene Accession Number	Gene ID OMIM	Primer Sequences (5′ → 3′)	Product (bp)
*ACTB*^a,b^ NM_001101	60 102630	sATGGCCACGGCTGCTTCCAGC asCATGGTGGTGCCGCCAGACAG	237
*β2-microglobulin (B2M)*^b^ NM_004048.2	567 109700	sTTGAAAAAGTGGAGCATTCAGA asTCGATCCCACTTAACTATCTTGG	155
*KCNJ1 (Kir1.1)*^a,b^ NM_153766.2	3758 600359	sGTGGAGGCACAGTCAAGGTT asCCACACAGGGAGTGTGATTG	202
*KCNJ2 (Kir2.1)*^a,b^ NM_000891.2	3759 600681	sCGGTGGATGCTGGTTATCTT asAAAACAGCAATTGGGCATTC	233
*KCNJ12 (Kir2.2)*^a,b^ NM_021012.4	3768 602323	sAGAAGAATGGCCAGTGCAAC asGCGATGACCCAGAAGATGAT	175
*KCNJ4 (Kir2.3)*^a,b^ NM_004981.1	3761 600504	sCCATCATCATTGTCCACGAG asGAAGACCACAGGCTCAAAGC	197
*KCNJ14 (Kir2.4)*^a,b^ NM_013348.3	3770 603953	sGATCGACTCTGCCAGTCCTC asTCCCTGGGACCTCATAAGTG	242
*KCNJ3 (Kir3.1)*^a,b^ NM_002239.3	3760 601534	sTGTGGAAACAACTGGGATGA asGGGACGACATGAGAAGCATT	203
*KCNJ6 (Kir3.2)*^a,b^ NM_002240.4	3763 600877	sGCTACCGGGTCATCACAGAT asATCAGGCACAGTTTCCCATC	196
*KCNJ9 (Kir3.3)*^a,b^ NM_004983.2	3765 600932	sGCTACCTGACGGACCTGTTC asAAGCCGTTGAGGTTGTTGAC	193
*KCNJ5 (Kir3.4)*^a,b^ NM_000890.3	3762 600734	sACCGATTCACACCAGTCCTC asATTCTGCTCAGCCTCTGCAT	212
*KCNJ10 (Kir4.1)*^a,b^ NM_002241.4	3766 602208	sCCAGGGATACGACGGCGGAGA asGAAACGAATGGTCTCAGCCCG	477
*KCNJ15 (Kir4.2)*^a^ NM_002243.4	3772 602106	sCCGTTCCATCACAGAGGAAT asTTCTGCTTGGTGATGACTGC	174
*KCNJ15 (Kir4.2)*^b^ NM_002243.4		sAGGTAGCCAATATGAGGAAGAGC asACAAGCTCAAACTCCTTCTCCTT	247
*KCNJ16 (Kir5.1)*^a,b^ NM_018658.2	3773 605722	sTCCACTGGAACATCTCACCA asACGTGCAGGATTCTCGAACT	232
*KCNJ8 (Kir6.1)*^a,b^ NM_004982.3	3764 600935	sGGAGGGAGGATGATGACAGA asTTTCCTCAGGTCACCCACTC	222
*KCNJ11 (Kir6.2)*^a,b^ NM_000525.3	3767 600937	sATCATCGTCATCCTGGAAGG asGGTGTTGCCAAACTTGGAG	162
*KCNJ13 (Kir7.1)*^a,b^ NM_002242.4	3769 603208	sTCACATGGATGGCAAACCTA asGCCAGAGGACTTGATGGTGT	225
*NFAT5*^b^ NM_006599.3	10725 604708	sTCACCATCATCTTCCCACCT asCTGCAATAGTGCATCGCTGT	174
*VEGFA_188, 164, 120_*^b^ NM_003376.5 NM_001287044.1 NM_001025370.2	7422 192240	sCCTGGTGGACATCTTCCAGGAGTA asCTCACCGCCTCGGCTTGTCACA	479; 407; 275

**Table 2:** Antibodies used in immunocytochemistry (ICC) and western blot (WB) analysis.

Method	Antibody	Source	Catalog	Dilution Concentration
ICC	rabbit anti-human Kir4.2	Sigma-Aldrich, Taufkirchen, Germany	HPA016702	1:100 2 µg/mL
	Alexa568-coupled goat anti-rabbit IgG	Invitrogen, Paisley, UK	A11036	1:500 4 µg/mL
WB	rabbit anti-human Kir4.2	Sigma-Aldrich	HPA016702	1:500 0.4 µg/mL
	mouse anti-human Kir4.2	Santa Cruz Biotechnology, Heidelberg, Germany	sc-376322	1:600 0.33 µg/mL
	rabbit anti-β-actin	Cell Signaling Technology, Frankfurt/M., Germany	8457	1:1000 37 ng/mL
	alkaline phosphatase-coupled goat anti-rabbit IgG	Cell Signaling Technology	7054	1:2000 35.5 ng/mL
	alkaline phosphatase-coupled goat anti-mouse IgG	Cell Signaling Technology	7056	1:2000 55.5 ng/mL

## Data Availability

The data are available on request from the corresponding author.

## Supplementary Materials

The following supporting information can be downloaded at: https://www.mdpi.com/article/10.3390/biom12060848/s1, Figure S1: Kir channel mRNA expression levels in acutely isolated and cultured human RPE cells.
