# Supplementary material for: Kir4.2 Potassium Channels in Retinal Pigment Epithelial Cells In Vitro: Contribution to Cell Viability and Proliferation, and Down-Regulation by Vascular Endothelial Growth Factor
Source: Biomolecules. 2022 Jun 18;12(6):848. doi: 10.3390/biom12060848 (PMC9220994; doi:10.3390/biom12060848)
Supplement: Supplementary file 1 [file biomolecules-12-00848-s001.zip › biomolecules-1735825-supplementary.pdf]

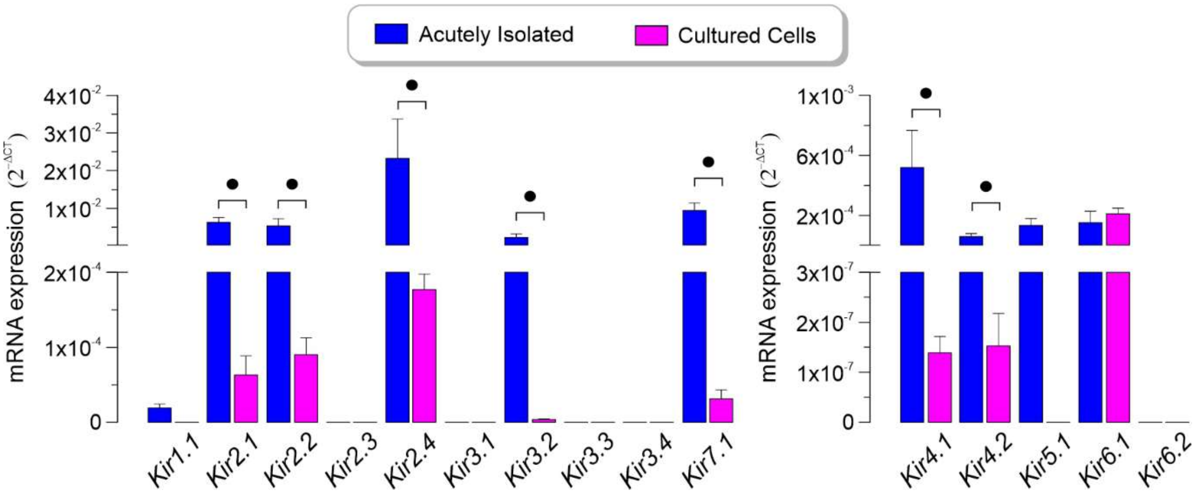

Supplemental Figure S1. Kir channel mRNA expression levels in acutely isolated and cultured human RPE cells. The expression levels were calculated using the formula $2^{-\Delta CT}$, with $\Delta CT = CT_{channel} - CT_{ACTB}$. Cells derived from 4 (cultured cells) to 6 eyes (acutely isolated cells) of different donors were used. Significant difference between acutely isolated and cultured cells: ●$P<0.05$.
